# Legionellosis and Lung Abscesses: Contribution of *Legionella* Quantitative Real-Time PCR to an Adapted Followup

**DOI:** 10.1155/2013/190183

**Published:** 2013-06-03

**Authors:** G. Descours, C. Tellini, C. Flamens, F. Philit, M. Celard, J. Etienne, G. Lina, S. Jarraud

**Affiliations:** ^1^CIRI, International Center for Infectiology Research, Université de Lyon, Lyon, France; ^2^Inserm, U1111, Lyon, France; ^3^Ecole Normale Supérieure de Lyon, Lyon, France; ^4^Université Lyon 1, Centre International de Recherche en Infectiologie, Lyon, France; ^5^CNRS, UMR5308, Lyon, France; ^6^Hospices Civils de Lyon, Groupement Hospitalier Est, Legionella National Reference Center, East Biology & Pathology Center, 59 Boulevard Pinel, 69677 Bron, France; ^7^Centre Hospitalier Pierre Oudot, Biology Laboratory, 30 Avenue du Medipole, 38317 Bourgoin-Jallieu, France; ^8^Hospices Civils de Lyon, Groupement Hospitalier Est, Department of Anesthesiology and Intensive Care, Louis Pradel Hospital, 28 Avenue Doyen Lépine, 69677 Bron, France; ^9^Hospices Civils de Lyon, Groupement Hospitalier Est, Department of Respiratory Medicine, Louis Pradel Hospital, 28 Avenue Doyen Lépine, 69677 Bron, France

## Abstract

We report a case of severe Legionnaires' disease (LD) complicated by a lung abscess in an immunocompetent patient who required ECMO therapy and thoracic surgery. The results of repeated *Legionella* quantitative real-time PCR performed on both sera and respiratory samples correlated with the LD severity and the poor clinical outcome. Moreover, the PCR allowed for the detection of *Legionella* DNA in the lung abscess specimen, which was negative when cultured for *Legionella*. This case report provides a logical basis for further investigations to examine whether the *Legionella* quantitative PCR could improve the assessment of LD severity and constitute a prognostic marker.

## 1. Introduction 

The detection of *Legionella* DNA by PCR is a criterion for the diagnosis of Legionnaires' disease (LD), but the contribution of repeated *Legionella* quantitative real-time PCR after diagnosis has not been evaluated so far. Here, we described a case of severe LD in an immunocompetent patient monitored by *Legionella* quantitative PCR under antimicrobial therapy. The persistence of stationary PCR cycle thresholds allowed for the detection of a lung abscess.

## 2. Case Presentation

A 28-year-old male smoker without previous illness presented to the emergency department with sudden left thoracic pain and dyspnoea. On examination, the patient's temperature was 37.6°C, heart rate was 90 beats/min, blood pressure was 110/60 mm Hg, and oxygen saturation was 93% on room air. The chest examination revealed crackles to the left upper lobe. The remainder of the examination was normal, and the patient was admitted to the short stay unit. Initial laboratory investigations revealed the following values: leucocyte count, 13,900 cells/mm^3^; 85% polymorphonuclear neutrophils; sodium, 136 mmol/L; potassium, 3.9 mmol/L; CRP, 36 mg/L; D-dimer under a positivity threshold; and normal cardiac, renal, and liver function tests. A chest X-ray and a thoracic computed tomography scan revealed a large left lower lobe compression. *Streptococcus pneumoniae* community-acquired pneumonia (CAP) was suspected, and the patient started receiving oral amoxicillin therapy according to the current recommendations [1]. 

On hospital day 3 and under increasing thoracic pain, the patient was transferred to the pneumology unit, where he rapidly deteriorated with a progressive cough that produced whitish sputum, left pleural pain requiring morphine administration, and a temperature spike to 40.5°C. On hospital day 4, his vital signs revealed a heart rate of 123 beats/min, a blood pressure of 118/69 mm Hg, a respiratory rate of 38 breaths/min, and an oxygen saturation of 89% on 10 L oxygen supply. The leucocyte count was 2.8 cells/mm^3^, CRP was 323 mg/L, natremia was 130 mmol/L, and phosphatemia was 0.64 mmol/L. Sputum was collected for microbiological investigations. Although a *Legionella* urinary antigen test was prescribed, no urine was ever collected. The antibiotic therapy was enlarged to intravenous amoxicillin/clavulanic acid and spiramycin, and the patient was transferred to the intensive care unit (ICU), where he rapidly deteriorated and required intubation. On day 5, the sputum indicated no respiratory pathogens after 24 hours of culturing on standard media. Analysis of the arterial blood gas indicated severe acute respiratory failure with a pH of 7.20, a CO_2_ pressure of 55.7 mm Hg, and an O_2_ pressure of 75.9 mm Hg on an FiO_2_, fraction of inspired oxygen, of 100%. The CRP was 446 mg/L, and the leucocyte count decreased to 0.8 cells/mm^3^. In spite of the antimicrobial therapy, which was switched to ceftriaxone, rovamycin, and levofloxacin, his temperature remained 39.5°C. He was then transferred to the cardiology ICU.

A few hours later, a laboratory examination revealed cardiac failure with a troponin of 0.19 *μ*g/L (normal range, <0.1 *μ*g/L). An ultrasonic cardiograph indicated that the left ventricular ejection fraction decreased to 30%. Venoarterial extracorporeal membrane oxygenation (VAECMO) therapy was initiated. The *Legionella* urine antigen (BinaxNOW *Legionella*, Alere SAS, Jouy-en-Josas, France) test was positive, and the antibiotic regimen was switched to erythromycin and levofloxacin. Using a serum specimen, the *Legionella* real-time PCR (PCR *L. pneumophila*, Diagenode, Evry, France) was also positive (Table 1).

On day 7, his leucocyte count was 14 cells/mm^3^. Bronchoalveolar lavages (BAL) and sputa cultures performed on day 4 grew *L. pneumophila* serogroup 1 (Lp1), which was typed as monoclonal antibody (mAb) 3/1-negative (Bellingham subtype) and sequence type (ST) 48 (Table 1) [2]. On day 13, a thoracic computed tomography scan likely evoked a lung abscess or necrosis on the left upper lobe, and rifampicin was added to the antibiotic regimen.

The patient's pulmonary functions progressively improved, and the VAECMO was discontinued 22 days later. However, the patient was still presenting temperature spikes. The chest X-ray and thoracic computed tomography scan demonstrated no improvement, and repeated BAL cultures indicated a persistent *Legionella pneumophila* infection (Table 1). The minimal extracellular concentrations inhibiting intracellular growth (MIEC) for erythromycin, levofloxacin, and rifampicin were determined on *Legionella* strains isolated from successive BAL, but no antibiotic resistance was observed (MIEC: erythromycin, 0.125 mg/L; levofloxacin, 0.016 mg/L; and rifampicin, 0.001 mg/L) [3]. In addition, the real-time PCR performed on the BAL demonstrated stationary cycle thresholds (Ct), reflecting stationary *Legionella* DNA amounts (Table 1).

**Figure 1:**
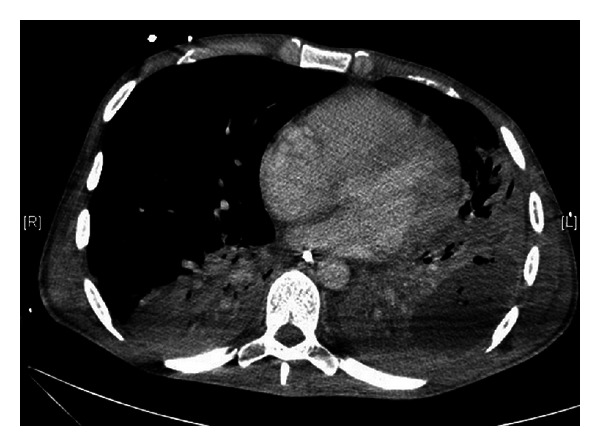
Thoracic CT scan revealing a voluminous lung abscess on the left pulmonary upper lobe on hospital day 35.

On day 34, a thoracic CT scan revealed a voluminous lung abscess on the left pulmonary upper lobe (Figure 1). An anaerobic BAL culture isolated *Fusobacterium nucleatum*, and nitroimidazole was added to the antibiotic regimen. The abscess was resected one week later (day 42) and revealed many leucocytes, and a culture was positive for *F. nucleatum*. Although the abscess culture was negative for *L. pneumophila*, the *Legionella* real-time PCR was positive (Table 1). The surgery allowed for a rapid clinical improvement. The fever resolved within 6 days, and mechanical ventilation was successfully discontinued 20 days after the surgery (day 62). The patient was discharged to the pneumology unit, where he completed a 7-week course of levofloxacin and nitroimidazole therapy. The CT scan indicated a complete resolution of the lung abscess, and the patient recovered completely. Water samples from the patients' home were investigated, but the source of the infection was not determined. The *Legionella* urinary antigen still tested positive one year later, which has been described in patients with a long-term defervescence of fever (Table 1) [4]. The patient remained well at the time of the follow-up examination at 2 years.

## 3. Discussion

 In this paper, we have described a case of severe Legionnaires' disease (LD), complicated by a lung abscess in an immunocompetent patient who required ECMO therapy and thoracic surgery. Few cases of acute respiratory failure due to* Legionella *and requiring ECMO therapy have been reported [5–7]. In such patients, Ichiba et al. described a survival rate limited from 25% to 53.8% [6]. The severity of this reported case might be attributed to a delayed diagnosis, and thus a delayed initiation of the antibiotic treatment, although guidelines for the management of CAP were followed [1, 8]. In this case, spiramycin was added on hospitalisation day 4 when the patient deteriorated. A specific anti-*Legionella* therapy combining a macrolide and fluoroquinolone was initiated on day 5 when the *Legionella *antigenuria test was positive. The severe leucopenia that developed during the first five days may also have contributed to the severity of the infection. Leucopenia has rarely been described during LD and has never been associated with a severe infection [9, 10]. However, the fundamental role of neutrophils in the clearance of *Legionella* from the lungs has been demonstrated with mouse studies [11]. The isolation of a mAb 3/1-negative *L. pneumophila* strain is unusual in an immunocompetent and nonhospitalised patient because the lipopolysaccharide reactivity pattern of mAb 3/1 mediates the virulence of *L. pneumophila *[2, 12]. MAb 3/1-negative strains have a high incidence among hospitalised and immunocompromised patients, whereas immunocompetent patients are mainly infected by mAb 3/1-positive strains. The isolation of a mAb 3/1-negative strain from this patient warranted an investigation of his cellular immunity, which was, however, unaffected.

 The microbiological results revealed a gradual decrease in the *Legionella* quantitative culture from the BAL according to the time (from day 4 to day 39, Table 1). Conversely, the real-time PCR Ct, which was inversely proportional to the amount of *Legionella* DNA present in the BAL sample as well as in the serum samples, increased over time (from day 5 to day 72 and from day 6 to day 26, resp.). These results demonstrate that repeated quantitative *Legionella* PCR from BAL and serum samples allows for a much more rapid and accurate surveillance of severe LD patients in comparison to conventional culturing methods. Moreover, the real-time PCR allowed for the detection of high *Legionella* DNA load in the lung abscess specimen, which was negative when cultured for *Legionella*. The persistence of viable but nonculturable (VBNC) *Legionella* in the lung abscess was considered, thus, the antibiotic treatment was not discontinued. The true incidence of *L. pneumophila* lung abscesses might be higher than suggested by culture investigations due to the difficulty involved in isolating the organism.

 The *Legionella* quantitative real-time PCR results correlated with the poor clinical outcome of the patient. *Legionella* DNA can be detected in serum within the first two weeks after the onset of symptoms [13–15]. Nevertheless, positive results on the first available serum sample are related to the severity of the LD at the time of diagnosis, and persisting positive results over two weeks are reported in less than 5% of patients [15–17]. In parallel, a high DNA load from the BAL at the time of admission also correlates with the severity of the LD, the need for hospitalisation in an ICU, and the duration of the hospitalisation [18]. Moreover, PCR on respiratory samples usually reaches its detection limit within the first week of the antimicrobial therapy, which was not observed with this patient [19]. As described by Diederen et al. on sera, our results illustrate that quantitative *Legionella* PCR on both sera and respiratory samples may allow for the assessment of the LD severity and may be a valuable tool to monitor the effects of antimicrobial therapies [20].

The infection was not resolved by antibiotic therapy and required surgery, likely because the therapeutic antibiotic levels did not reach within the abscess. Only a few cases of lung abscesses complicating LD have been described in immunocompromised patients [21–23]. The persistence of positive *Legionella* cultures and stationary PCR Ct may systematically investigate the presence of abscesses that have been described as factors associated with *Legionella*-related mortality [24, 25].

In summary, the conventional culturing of respiratory samples is essential for the isolation of *Legionella* strains and allows for epidemiological investigations. Nevertheless, repeated *Legionella* quantitative real-time PCR on serum and respiratory samples appears to be highly beneficial for the evaluation of LD severity and its prognosis and for monitoring severely ill patients under antimicrobial therapy. In this paper, the persistence of stationary PCR Ct correlated with poor clinical outcome and allowed for the detection of a lung abscess in an immunocompetent patient. This provides a logical basis for further investigations to examine whether the *Legionella* quantitative PCR could improve the assessment of LD severity and constitute a prognostic marker.

## Figures and Tables

**Table 1:** Microbiological results for *Legionella* during the patient's hospitalisation.

	Day after admission (D)																
	D4	D5	D6	D11	D14	D17	D22	D24	D26	D29	D34	D39	D42 Surgery	D43	D54	D72	D380
*Legionella* urine antigen test		+															+

Axenic culture (number of colonies on an agar plate)																	
BAL specimen*	+ (>500)	+ (>500)	+ (>500)			+ (26)	+ (5)	+ (10)	+ (1)	+ (8)	+ (3)	+ (1)	−	−	−	−	
Lung abscess specimen													−	−			

*L. pneumophila* PCR Ct																	
BAL specimen		18.9	20.0			21.3	26.1	23.1	25.0	24.3	25.0	25.3	22.7	27.4	34.1	>45	
Lung abscess specimen													17.3	16.0			
Serum specimen			33.2	30.9	32.7		36.1		>45								

BAL, bronchoalveolar lavage; Ct, cycle threshold; +, positive; and −, negative.

*except on D4: sputum specimen.
